# Ticagrelor vs. Clopidogrel in Acute Coronary Syndrome Patients With Chronic Kidney Disease After New-Generation Drug-Eluting Stent Implantation

**DOI:** 10.3389/fcvm.2021.707722

**Published:** 2022-01-10

**Authors:** Ji Woong Roh, Seung-Jun Lee, Byeong-Keuk Kim, Sung-Jin Hong, Hee-Yeol Kim, Chul-Min Ahn, Deok-Kyu Cho, Jung-Sun Kim, Young-Guk Ko, Donghoon Choi, Myeong-Ki Hong, Yangsoo Jang

**Affiliations:** ^1^Division of Cardiology, Department of Internal Medicine, Yonsei University College of Medicine, Yongin Severance Hospital, Yongin, South Korea; ^2^Department of Cardiology, The Catholic University College of Medicine, Bucheon St. Mary's Hospital, Bucheon, South Korea; ^3^Division of Cardiology, Department of Internal Medicine, Yonsei University College of Medicine, Severance Cardiovascular Hospital, Seoul, South Korea; ^4^Department of Cardiology, CHA Bundang Medical Centre, CHA University, Seongnam, South Korea

**Keywords:** ticagrelor, clopidogrel, acute coronary syndrome, renal insufficiency, drug eluting stents

## Abstract

**Background:** The impact of ticagrelor-based dual antiplatelet therapy (DAPT) on acute coronary syndrome (ACS) in patients with chronic kidney disease (CKD) remains unclear.

**Methods:** Data on a total of 1,067 ACS patients with CKD including end-stage renal disease (ESRD) who underwent new-generation drug-eluting stent implantation were extracted from a multicenter registry. This study aimed to compare outcomes of patients treated with ticagrelor- (*n* = 449) and those treated with clopidogrel-based (*n* = 618) DAPT. Outcomes of interest included major adverse cardiac and cerebrovascular events (MACCEs) and bleeding (Bleeding Academic Research Consortium grade 3 or 5) at 12 months. Propensity-score matching (346 pairs) analysis was performed.

**Results:** The patients with ESRD showed the highest MACCE and bleeding rates (*P* < 0.001). There was no difference in the rate of MACCEs between the treatment groups (7.8% vs. 8.4%; hazard ratio [HR] = 0.95, 95% confidence interval [CI] = 0.56–1.61, *P* = 0.855); however, a trend toward an increased bleeding rate was observed in the ticagrelor-based DAPT group (6.8% vs. 3.8%, HR = 1.84, 95% CI = 0.93–3.63, *P* = 0.079). Among patients with CKD stage III/IV but without ESRD (277 pairs), the ticagrelor-based DAPT group showed a reduced MACCE rate (3.6% vs. 8.7%, HR = 0.41, 95% CI = 0.19–0.86, *P* = 0.018) and a similar bleeding rate (5.1% vs. 3.2%, HR = 1.61, 95% CI = 0.70–3.71, *P* = 0.267), compared with those of the clopidogrel-based DAPT group.

**Conclusion:** The effects of ticagrelor-based DAPT on ischemic and bleeding outcomes of ACS patients with CKD varied according to CKD stage; in ACS patients with CKD without ESRD, ticagrelor-based DAPT reduced MACCE risk without increasing bleeding risks, relative to those observed with clopidogrel-based DAPT.

## Introduction

Several studies suggest that ticagrelor has superior efficacy over clopidogrel in reducing the risk of major adverse cardiovascular events in patients with acute coronary syndrome (ACS) (1–3). Current guidelines recommend dual antiplatelet therapy (DAPT) with a combination of aspirin and a potent P2Y_12_ inhibitor for 12 months in the era of new-generation drug-eluting stent (DES) (4–6). However, although chronic kidney disease (CKD), including end-stage renal disease (ESRD), is a well-documented risk factor for recurrent ischemic major adverse cardiac and cerebrovascular events (MACCEs) and bleeding events (7, 8), the optimal antiplatelet strategy for patients with CKD remains unclear due to the lack of clinical trial-based evidence (4, 8). The present study aimed to compare clinical outcomes of patients with ACS and CKD treated with the new-generation DES, stratified by CKD stage, and dichotomized based on P2Y_12_ inhibitor type used in DAPT (ticagrelor vs. clopidogrel).

## Materials and Methods

### Study Design and Patient Selection

**Figure 1:**
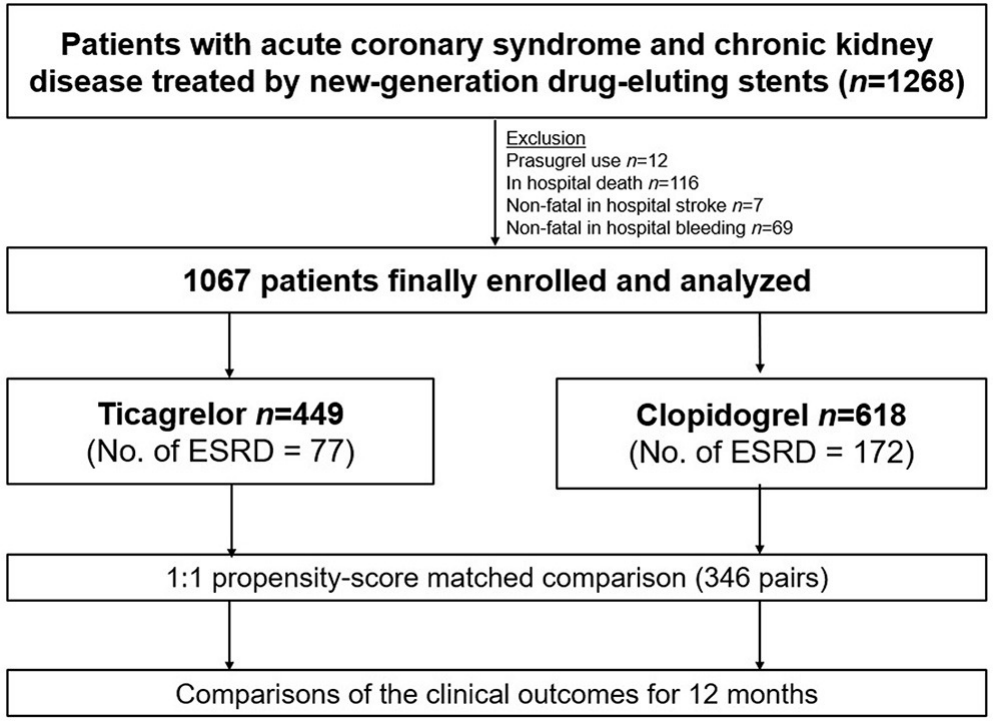
Study flow diagram. ESRD, End Stage Renal Disease.

A study flow diagram is presented in Figure 1. Between 2013 and 2019, data on a total of 1,268 patients with CKD, including those with ESRD, who presented with ACS and underwent percutaneous coronary intervention (PCI) using new-generation DES were obtained from the prospective Korean multicenter angioplasty team (NCT03908463) and Bucheon St. Mary's CKD registry. After excluding cases of prasugrel use, in-hospital death, stroke, or bleeding, a total of 1,067 patients with CKD, including those with ESRD (*n* = 249 [23.3%]), were included and dichotomized according to the type of P2Y_12_ receptor inhibitor prescribed at discharge into the ticagrelor- (*n* = 449) and clopidogrel-based (*n* = 618) DAPT groups. The study protocol was approved by the institutional review board at each participating site.

### Definition

CKD was defined as an estimated glomerular filtration rate (eGFR) of <60 mL/min/1.73 m^2^ (9, 10). The patients with acute renal failure were excluded. It was calculated using the Chronic Kidney Disease Epidemiology Collaboration equation in accordance with the National Kidney Foundation guidelines (11) using the latest laboratory values for the renal function measured before the index PCI. CKD was classified as stage IIIa, IIIb, and IV, given eGFR values in the range of 45–59, 30–44, and 15–29 mL/min/1.73 m^2^; ESRD was defined as eGFR of <15 mL/min/1.73 m^2^.

The primary endpoint was the incidence of MACCEs, defined as the composite of cardiac death, myocardial infarction (MI), stent thrombosis, and cerebrovascular accidents (CVA) at 12 months. The secondary endpoints were rates of major bleeding (grade 3 or 5, according to the Bleeding Academic Research Consortium [BARC] criteria) (12), and of net adverse clinical events, including both MACCEs and major bleeding events. All clinical events were defined according to the Academic Research Consortium (13). All deaths were considered cardiac deaths unless a definite non-cardiac cause was established. Myocardial infarction (MI) after discharge from the hospital was defined as clinical symptoms, or electrocardiography changes combined with a creatine kinase MB fraction above the upper normal limits or a troponin T or troponin I level >99th percentile of the upper normal limit (14). Stent thrombosis was defined as definite or possible thrombosis. CVA was defined as the occurrence of any focal neurological deficit confirmed by a neurologist with brain imaging studies. Target vessel revascularization is defined as a repeat PCI or bypass surgery of the target vessel with either: (1) ischemia symptoms or a positive stress test with angiographic diameter stenosis >50%; or (2) angiographic diameter stenosis >70% without ischemia symptoms or a positive stress test. Follow-up assessment was performed at 1, 3, 6, 9, and 12 months (365 ± 30 days) either by a clinical visit or telephone interview.

### Statistical Analysis

Categorical variables were reported as frequencies and percentages; continuous variables were reported as means ± standard deviations. Continuous variables were compared with the student *t*-test, and categorical variables were compared using the chi-square test or Fisher exact test, as appropriate. To minimize selection bias, propensity-score matching was performed using a multivariable logistic regression model, in which treatment status was the dependent variable and baseline, clinical, angiographic, and procedural characteristics, including age, sex, hypertension, diabetes, previous MI, CKD stage, ESRD, and clinical presentation, were the independent variables. Thereafter, the patients receiving ticagrelor-based DAPT were matched 1:1 with those receiving clopidogrel-based DAPT using propensity scores with the nearest available pair-matching method; a total of 346 matched pairs were generated. Hazard ratios for the primary and secondary endpoints were calculated with the multivariable Cox proportional hazards model and reported with the corresponding 95% confidence intervals. Subgroup analyses were performed by including an interaction term in the proportional hazards model. To determine the predictors of MACCEs, multivariate Cox regression analysis with propensity score-matched patients with CKD (including all disease stages or excluding ESRD) was performed, including all variables significantly associated with the outcomes of interest (*P-*value of <0.1) in univariate analysis. The Kaplan-Meier method with the log-rank test was used to compare the cumulative rates of MACCEs and bleeding events among groups before and after propensity-score matching. All tests were 2-sided, and the results were considered statistically significant at *P*-values of <0.05. All analyses were performed with R software version 3.4.4 (R Foundation for Statistical Computing, Vienna, Austria).

## Results

The patients' baseline characteristics are presented in the Table 1. Patients in the ticagrelor-based DAPT group were more likely to be younger male smokers, have a higher body mass index, have a history of ST-elevation MI, cardiogenic shock before PCI, and multivessel PCI, and less likely to have hypertension or diabetes mellitus, or a history of previous PCI or bypass surgery, or present ESRD than were patients in the clopidogrel-based DAPT group. After propensity-score matching, there was no significant difference between the groups, except for the rates of multi-vessel PCI and types of DES implanted (Table 1).

**Table 1:** Baseline characteristics.

	**Crude analyses**			**Propensity-score matching**			
	**Ticagrelor-based DAPT (*N* = 449)**	**Clopidogrel-based DAPT (*N* = 618)**	***P*-value**	**Ticagrelor-based DAPT (*N* = 346)**	**Clopidogrel-based DAPT (*N* = 346)**	***P*-value**	**SMD**
Age, years	68.3 ± 10.9	70.4 ± 10.6	0.001	68.2 ± 11.0	69.8 ± 10.7	0.067	0.11
Male	355 (79.1%)	430 (69.6%)	0.001	264 (76.3%)	256 (74.0%)	0.538	0.05
Body mass index (kg/m^2^)	24.5 ± 3.5	24.0 ± 3.5	0.043	24.4 ± 3.5	23.9 ± 3.4	0.051	0.12
Hypertension	333 (74.2%)	499 (80.7%)	0.013	267 (77.2%)	279 (80.6%)	0.305	0.07
Diabetes mellitus	252 (56.1%)	385 (62.3%)	0.049	213 (61.6%)	209 (60.4%)	0.815	0.02
Dyslipidemia	264 (58.8%)	358 (57.9%)	0.825	212 (61.3%)	195 (56.4%)	0.217	0.09
Smoker	213 (47.4%)	251 (40.6%)	0.031	157 (45.4%)	151 (43.6%)	0.702	0.05
Previous PCI	80 (17.8%)	154 (24.9%)	0.007	72 (20.8%)	81 (23.4%)	0.464	0.06
Previous MI	40 (8.9%)	63 (10.2%)	0.551	37 (10.7%)	40 (11.6%)	0.809	0.04
Previous bypass surgery	6 (1.3%)	30 (4.9%)	0.003	6 (1.7%)	11 (3.2%)	0.326	0.07
Previous cerebrovascular accident	50 (11.1%)	93 (15.0%)	0.078	41 (11.8%)	55 (15.9%)	0.153	0.09
Congestive heart failure (Killip II–IV)	23 (5.1%)	33 (5.3%)	0.986	19 (5.5%)	18 (5.2%)	0.986	0.01
Stage of chronic kidney diseases			<0.001			0.446	0.06
Stage IIIa Stage IIIb	225 (50.1%) 93 (20.7%)	252 (40.8%) 111 (18.0%)		163 (47.1%) 62 (17.9%)	143 (41.3%) 70 (20.2%)		
Stage lV	54 (12.0%)	83 (13.4%)		45 (13.0%)	54 (15.6%)		
End stage renal disease	77 (17.1%)	172 (27.8%)	<0.001	76 (22.0%)	79 (22.9%)	0.855	0.03
Hemodialysis	65 (14.5%)	152 (24.6%)		64 (18.5%)	68 (19.7%)		
Peritoneal dialysis	12 (2.7%)	20 (3.2%)		12 (3.5%)	11(3.2%)		
Clinical presentation			<0.001			0.934	0.01
Unstable angina	102 (22.7%)	334 (54.0%)		102 (29.5%)	106 (30.6%)		
Non-ST-elevation MI	202 (45.0%)	243 (39.3%)		202 (58.4%)	200 (57.8%)		
ST-elevation MI	145 (32.3%)	41 (6.6%)		42 (12.1%)	40 (11.6%)		
Cardiogenic shock before PCI	34 (7.6%)	24 (3.9%)	0.013	20 (5.8%)	23 (6.6%)	0.753	0.03
Duration of dual antiplatelet therapy ≤ 6 months	136 (30.3%)	147 (23.8%)	0.083	99 (28.6%)	84 (24.3%)	0.479	0.05
Multi-vessel diseases	347 (77.3%)	491 (79.4%)	0.438	272 (78.6%)	281 (81.2%)	0.448	0.05
Treated vessel, left anterior descending artery	247 (55.0%)	342 (55.3%)	0.965	191 (55.2%)	187 (54.0%)	0.819	0.02
Long lesion (**≥**28 mm)	261 (58.1%)	339 (54.9%)	0.316	199 (57.5%)	191 (55.2%)	0.592	0.05
Small-vessel disease (**≤**2.75 mm)	163 (36.3%)	259 (41.9%)	0.074	145 (41.9%)	150 (43.4%)	0.758	0.03
Multi-vessel PCI	125 (27.8%)	103 (16.7%)	<0.001	107 (30.9%)	64 (18.5%)	<0.001	0.32
No. of treated lesion per vessel	1.21 ± 0.46	1.21 ± 0.42	0.953	1.19 ± 0.40	1.20 ± 0.45	0.896	0.02
No. of stents per lesion	1.22 ± 0.42	1.17 ± 0.38	0.084	1.23 ± 0.43	1.16 ± 0.37	0.028	0.15
Type of drug eluting stents			<0.001			<0.001	0.25
Sirolimus-eluting Zotalimus-eluting Everolimus-eluting	289 (64.4%) 70 (15.6%) 55 (12.2%)	183 (29.6%) 200 (32.4%) 106 (17.2%)		215 (62.1%) 64 (18.5%) 38 (11.0%)	86 (24.9%) 124 (35.8%) 64 (18.5%)		
Biolimus-eluting	13 (2.9%)	82 (13.3%)		11 (3.2%)	50 (14.5%)		
Others	22 (4.9%)	47 (7.5%)		18 (5.2%)	22 (6.4%)		
Total stented length per lesion, mm	28.3 ± 13.2	27.5 ± 13.5	0.359	28.8 ± 14.0	27.8 ± 13.9	0.331	0.07
Mean stent diameter, mm	3.1 ± 0.6	3.1 ± 0.5	0.260	3.1 ± 0.6	3.1 ± 0.7	0.218	0.09
Intra vascular ultrasound use	62 (13.8%)	65 (10.5%)	0.614	47 (13.6%)	39 (11.3%)	0.420	0.05

*Values are presented as number (%) or mean ± SD*.

*PCI, percutaneous coronary intervention; MI, myocardial infarction; SMD, standardized mean difference*.

**Figure 2:**
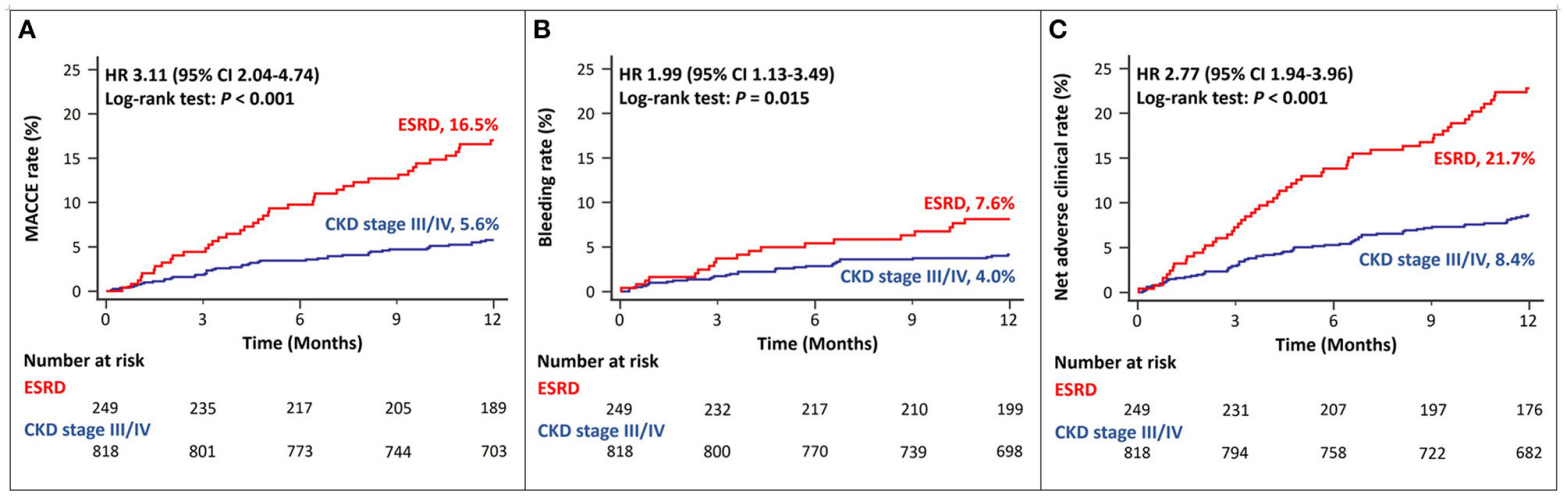
Twelve-month cumulative incidence of major adverse cardiac and cerebrovascular events **(A)**, bleeding **(B)**, or net adverse clinical **(C)** events, compared between CKD stage III/IV and ESRD. MACCE, Major Adverse Cardiac and Cerebrovascular Event; CKD, Chronic Kidney Disease; ESRD, End Stage Renal Disease.

Among patients with ACS and CKD, the rates of MACCEs differed according to the CKD stage. Patients with ESRD had a significantly higher rate of MACCEs than those with CKD stage III/IV (Figure 2A). The rates of bleeding events also differed among patients with different stages of CKD. Patients with ESRD showed higher rates of major bleeding events than those with CKD stage III/IV (Figure 2B). The rates of net adverse clinical events were also significantly higher in patients with ESRD than in those with CKD stage III, IV (Figure 2C).

**Figure 3:**
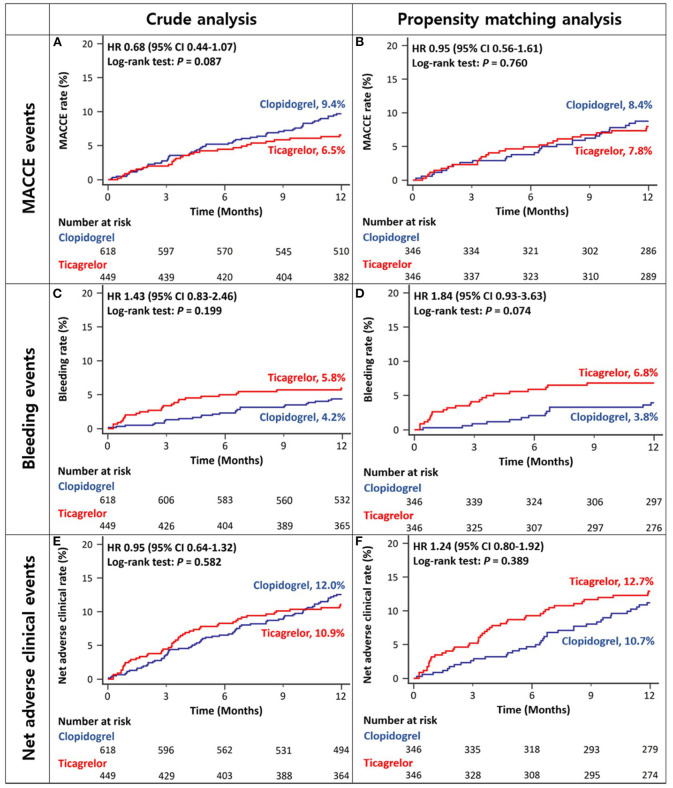
Twelve-month cumulative incidence of major adverse cardiac and cerebrovascular events **(A,B)**, bleeding **(C,D)**, or net adverse clinical **(E,F)** events among acute coronary syndrome patients with CKD in crude **(A,C,E)** and propensity-score matched analyses **(B,D,F)**. MACCE, Major Adverse Cardiac and Cerebrovascular Event; CKD, Chronic Kidney Disease.

There was no difference between ACS patients with CKD treated with ticagrelor- vs. those treated with clopidogrel-based DAPT in the rates of MACCEs at 12 months in either crude or propensity score-matched analysis (Figures 3A,B and Table 2). There was no significant between-group difference in the rates of bleeding events in crude analysis; however, in propensity score-matched analysis, the ticagrelor-based DAPT group showed a trend toward a higher bleeding rate than the clopidogrel-based DAPT group (Figures 3C,D and Table 2). There was no between-group difference in the rates of net adverse clinical events or MACCE components in either crude or propensity score-matched analysis (Figures 3E,F and Table 2). However, ticagrelor-based DAPT was associated with a higher rate of BARC type 2 bleeding than clopidogrel-based DAPT in both crude and propensity score-matched analyses (Table 2).

**Table 2:** Clinical outcomes at 12 months.

	**Crude analysis**				**Propensity-score matching analysis**			
**Overall CKD patients**	**Ticagrelor-based DAPT (*N* = 449)**	**Clopidogrel-based DAPT (*N* = 618)**	**HR (95% CI)**	***P*-value**	**Ticagrelor -based DAPT (*N* = 346)**	**Clopidogrel -based DAPT (*N* = 346)**	**HR (95% CI)**	***P*-value**
MACCE	29 (6.5%)	58 (9.4%)	0.68 (0.44–1.07)	0.095	27 (7.8%)	29 (8.4%)	0.95 (0.56–1.61)	0.855
Bleeding events (BARC type 3 or 5)	26 (5.8%)	26 (4.2%)	1.43 (0.83–2.46)	0.200	23 (6.8%)	13 (3.8%)	1.84 (0.93–3.63)	0.079
Net adverse clinical events	49 (10.9%)	74 (12.0%)	0.95 (0.64–1.32)	0.661	44 (12.7%)	37 (10.7%)	1.24 (0.80–1.92)	0.335
***Individual event***
All-cause death	31 (6.9%)	43 (7.0%)	0.99 (0.62–1.57)	0.967	29 (8.4%)	18 (5.2%)	1.62 (0.90–2.91)	0.109
Cardiac death	13 (2.9%)	26 (4.2%)	0.69 (0.35–1.34)	0.274	11 (3.2%)	10 (2.9%)	1.10 (0.47–2.60)	0.820
Non-cardiac death	18 (4.0%)	17 (2.8%)	1.45 (0.75–2.82)	0.271	18 (5.2%)	8 (2.3%)	2.25 (0.98–5.18)	0.056
Myocardial infarction	19 (4.2%)	28 (4.5%)	0.94 (0.53–1.69)	0.843	17 (4.9%)	17 (4.9%)	1.01 (0.52–1.98)	0.978
Stent thrombosis	6 (1.3%)	11 (1.8%)	0.75 (0.28–2.03)	0.569	4 (1.2%)	5 (1.4%)	0.79 (0.21–2.94)	0.726
Cerebrovascular accident	8 (1.8%)	15 (2.4%)	0.73 (0.31–1.73)	0.478	8 (2.3%)	6 (1.7%)	1.34 (0.46–3.85)	0.593
Ischemic	3 (0.7%)	10 (1.6%)			3 (0.9%)	2 (0.5%)		
Hemorrhagic	5 (1.1%)	5 (0.8%)			5 (1.4%)	4 (1.2%)		
Target-vessel revascularization	10 (2.2%)	29 (4.7%)	0.47 (0.23–0.97)	0.051	10 (2.9%)	16 (4.6%)	0.62 (0.28–1.36)	0.228
BARC type 2	15 (3.3%)	7 (1.1%)	3.34 (1.34–8.34)	0.010	12 (3.5%)	4 (1.2%)	3.67 (1.13–11.9)	0.030
BARC type 2, 3, or 5	41 (9.1%)	33 (5.3%)	1.81 (1.14–2.87)	0.012	35 (10.1%)	17 (4.9%)	2.23 (1.24–4.00)	0.007
**CKD stage III, IV with ESRD excluded**	**Ticagrelor-based DAPT** **(*****N*** **=** **372)**	**Clopidogrel-based DAPT** **(*****N*** **=** **446)**	**HR (95% CI)**	***P*** **-value**	**Ticagrelor-based DAPT** **(*****N*** **=** **277)**	**Clopidogrel -based DAPT (*****N*** **=** **277)**	**HR (95% CI)**	***P*** **-value**
MACCE	13 (3.3%)	33 (7.4%)	0.47 (0.25–0.89)	0.020	10 (3.6%)	24 (8.7%)	0.41 (0.19–0.86)	0.018
Bleeding events	18 (4.9%)	15 (3.3%)	1.49 (0.75–2.95)	0.258	14 (5.1%)	9 (3.2%)	1.61 (0.70–3.71)	0.267
(BARC type 3 or 5)								
Net adverse clinical events	27 (7.3%)	42 (9.4%)	0.77 (0.48–1.25)	0.296	21 (7.6%)	29 (10.5%)	0.72 (0.41–1.26)	0.255
***Individual event***
All-cause death	13 (3.5%)	24 (5.4%)	0.64 (0.33–1.27)	0.201	12 (4.3%)	17 (6.1%)	0.70 (0.34–1.47)	0.349
Cardiac death	3 (0.8%)	13 (2.9%)	0.28 (0.08–0.98)	0.046	3 (1.1%)	10 (3.6%)	0.31 (0.08–1.11)	0.072
Non-cardiac death	10 (2.7%)	11 (2.5%)	1.08 (0.46–2.53)	0.869	9 (3.2%)	7 (2.5%)	1.27 (0.47–3.40)	0.640
Myocardial infarction	6 (1.6%)	15 (3.4%)	0.48 (0.19–1.23)	0.124	5 (1.8%)	14 (5.1%)	0.35 (0.13–0.98)	0.046
Stent thrombosis	3 (0.8%)	6 (1.3%)	0.60 (0.15–2.38)	0.464	3 (1.1%)	5 (1.8%)	0.60 (0.14–2.50)	0.481
Cerebrovascular accident	7 (1.9%)	11 (2.5%)	0.76 (0.30–1.96)	0.570	5 (1.8%)	6 (2.2%)	0.83 (0.25–2.72)	0.758
Ischemic	3 (0.8%)	8 (1.8%)			2 (0.7%)	4 (1.5%)		
Hemorrhagic	4 (1.1%)	3 (0.7%)			3 (1.1%)	2 (0.7%)		
Target-vessel revascularization	3 (0.8%)	19 (4.3%)	0.19 (0.06–0.63)	0.007	3 (1.1%)	10 (3.6%)	0.30 (0.08–1.09)	0.067
BARC type 2	9 (2.4%)	2 (0.4%)	7.37 (1.46–37.1)	0.015	7 (2.5%)	2 (0.7%)	5.13 (0.94–28.1)	0.059
BARC type 2, 3, or 5	27 (7.3%)	17 (3.8%)	2.05 (1.11–3.78)	0.022	21 (7.6%)	11 (4.0%)	2.11 (1.01–4.44)	0.048

*Values are presented as numbers and the cumulative event rates (%)*.

*CKD, chronic kidney disease; MACCE, major adverse cardiac and cerebrovascular events; BARC, bleeding academic research consortium; CI, confidence interval; HR, hazards ratio*.

**Figure 4:**
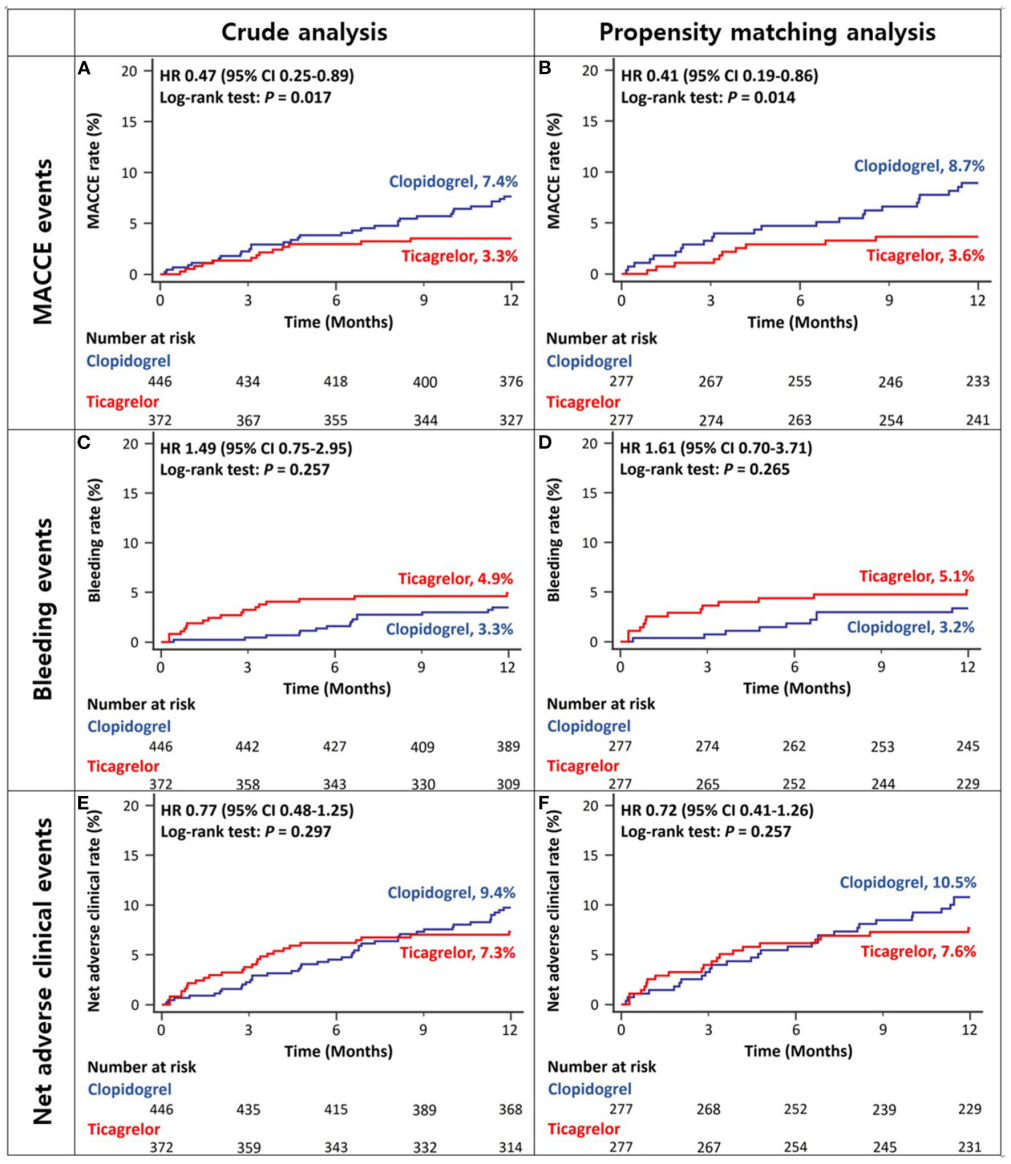
Twelve-month cumulative incidence of major adverse cardiac and cerebrovascular events **(A,B)**, bleeding **(C,D)**, or net adverse clinical **(E,F)** events in patients with CKD stage III/IV without ESRD in crude **(A,C,E)** and propensity-score matched analyses **(B,D,F)**. MACCE, Major Adverse Cardiac and Cerebrovascular Event; CKD, Chronic Kidney Disease; ESRD, End Stage Renal Disease.

After excluding the patients with ESRD, the 12-month rates of MACCEs in ACS patients with CKD stage III/IV were lower in the ticagrelor-based DAPT group than in the clopidogrel-based DAPT group in both crude and propensity score-matched analyses (Figures 4A,B and Table 2). There was no significant between-group difference in the rates of bleeding (Figures 4C,D and Table 2) or net adverse clinical events (Figures 4E,F and Table 2) in either crude or propensity score-matched analyses. In addition, the rates of cardiac death or large-vessel revascularization were lower and those of BARC 2 or 2, 3, or 5 bleeding were higher in the ticagrelor-based DAPT group than in the clopidogrel-based DAPT group in crude analysis. After propensity score matching, the rate of MI was lower and that of BARC 2, 3, or 5 bleeding was higher in the ticagrelor-based DAPT group than in the clopidogrel-based DAPT group (Table 2).

**Figure 5:**
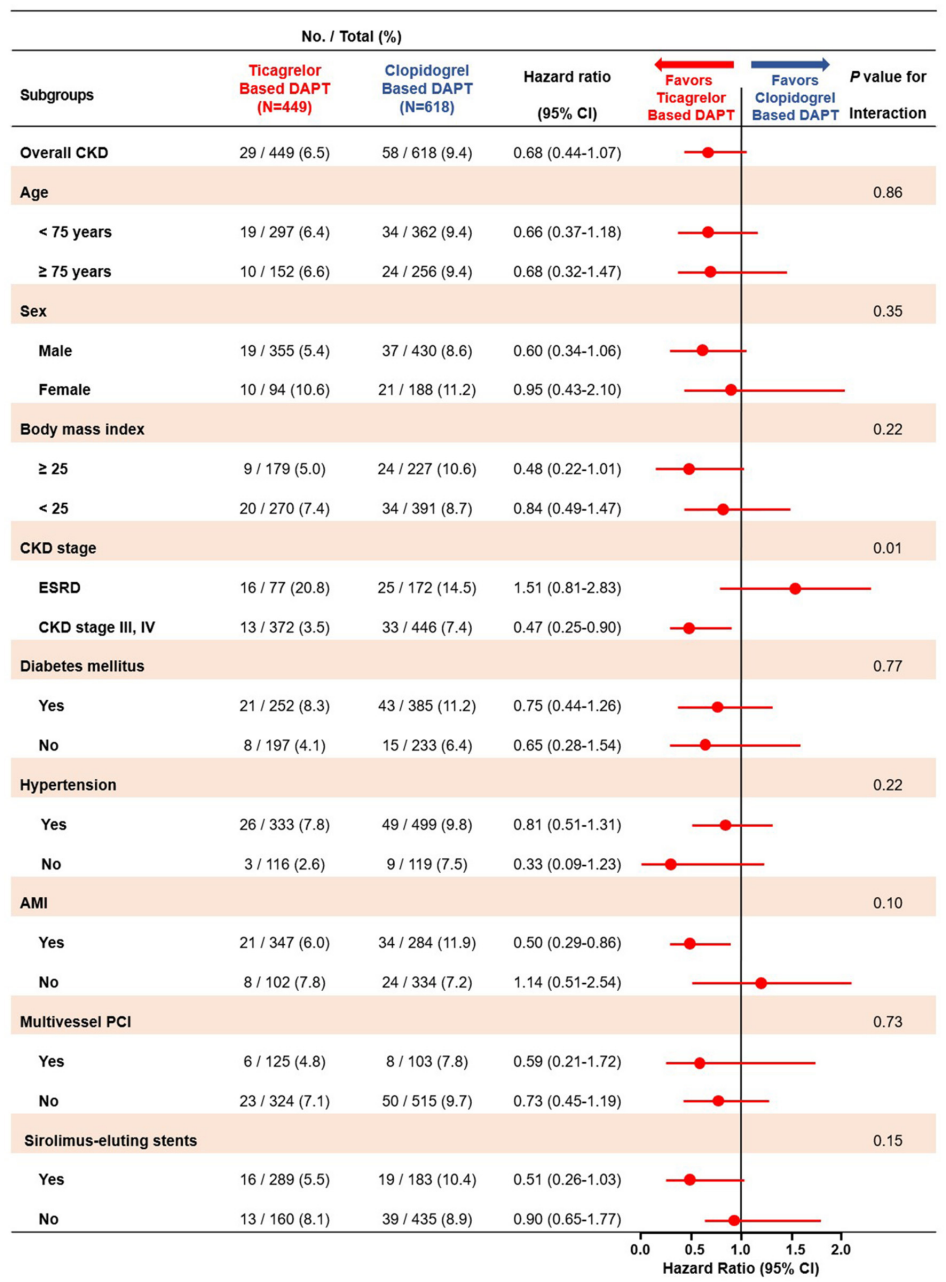
Subgroup analyses of major adverse cardiac and cerebrovascular events between the ticagrelor- and clopidogrel-based DAPT groups. MACCE, Major Adverse Cardiac and Cerebrovascular Event; DAPT, Dual Anti Platelet Therapy; CKD, Chronic Kidney Disease; ESRD, End Stage Renal Disease; AMI, Acute Myocardial Infarction; PCI, Percutaneous Coronary Intervention.

In subgroup analyses for MACCE, among the patients with CKD stage III/IV, those in the ticagrelor-based DAPT group had a more favorable outcomes than did those in the clopidogrel-based DAPT group with significant interaction. Except CKD stage, there was no interaction between the various subgroup categories (Figure 5). On subgroup analyses regarding the bleeding events, there was no significant interaction between the various subsets (Supplementary Figure 1).

Among patients with ESRD (*n* = 249), there was no significant difference in the rates of MACCEs or major bleeding between the ticagrelor- and clopidogrel-based DAPT groups (Supplementary Figures 2A,B and Supplementary Table 2). However, there was a trend toward a higher rate of net adverse clinical events in the ticagrelor-based DAPT group than in the clopidogrel-based DAPT group (Supplementary Figure 2C and Supplementary Table 2).

Propensity score-matched multivariate analyses of the overall CKD sample revealed that previous PCI or bypass surgery, congestive heart failure, ESRD, and the presence of a long lesion (≥28 mm) were significant predictors of MACCEs. Meanwhile, among patients with CKD stage III/IV but without ESRD, the use of ticagrelor reduced the risk of MACCE, while age of ≥75 years and a history of bypass surgery increased this risk (Table 3).

**Table 3:** Predictors for the occurrence of major adverse cardiac and cerebrovascular events.

**Overall CKD patients (N = 692)**	**Univariate analysis**		**Multivariate analysis**	
	**HR (95% CI)**	***P*-value**	**HR (95% CI)**	***P*-value**
Diabetes mellitus	2.19 (1.18–4.08)	0.013	1.29 (0.67–2.49)	0.439
Previous percutaneous coronary intervention	2.44 (1.43–4.17)	0.001	1.97 (1.11–3.50)	0.021
Previous bypass surgery	6.16 (2.79–13.60)	<0.001	4.58 (1.95–10.76)	<0.001
Congestive heart failure (Killip II–IV)	3.87 (1.90–7.91)	<0.001	3.42 (1.59–7.37)	0.002
ESRD	3.50 (2.07–5.91)	<0.001	2.51 (1.45–4.35)	0.001
Multivessel disease	3.40 (1.23–9.41)	0.018	2.03 (0.71–5.79)	0.186
Left main disease	2.61 (1.38–4.95)	0.003	1.23 (0.61–2.47)	0.572
Long lesion (≥28 mm)	1.69 (0.97–2.97)	0.066	1.75 (1.01–3.09)	0.049
Use of the ticagrelor	0.95 (0.56–1.61)	0.855		
**CKD stage III, IV with ESRD excluded (N** **=** **554)**	**Univariate analysis**		**Multivariate analysis**	
	**HR (95% CI)**	***P*** **-value**	**HR (95% CI)**	***P*** **-value**
Age ≥75 years	2.58 (1.26–5.29)	0.010	2.18 (1.05–4.52)	0.036
Male	0.44 (0.22–0.87)	0.018	0.54 (0.27–1.09)	0.085
Diabetes mellitus	2.15 (1.00–4.61)	0.049	1.82 (0.83–3.97)	0.135
Previous bypass surgery	6.77 (2.38–19.21)	<0.001	5.12 (1.79–14.64)	0.002
Multivessel disease	3.05 (0.93–9.98)	0.065	2.70 (0.81–8.98)	0.107
Use of the ticagrelor	0.41 (0.19–0.86)	0.018	0.46 (0.22–0.95)	0.037

*CKD, chronic kidney disease; ESRD, end staged renal disease*.

## Discussion

The major findings of the present study include the following: First, among the ACS patients with CKD who underwent new generation DES implantation and were safely discharged, the rates of MACCEs or bleeding events differed according to the severity of renal impairment. Patients with ESRD had higher rates of MACCEs, bleeding, and net adverse clinical events than those with CKD stage III/IV. Second, there was no significant difference in the rates of MACCEs, bleeding, or net adverse clinical events between patients treated with ticagrelor- and those treated with clopidogrel-based DAPT. Third, among patients with CKD stage III/IV without ESRD, ticagrelor-based DAPT treatment was associated with a decreased rate of MACCEs, and a similar rate of bleeding compared with clopidogrel-based DAPT treatment. Fourth, the use of ticagrelor was associated with reduced risk of MACCEs in patients with CKD stage III/IV without ESRD, suggesting that the use of ticagrelor in this patient group may improve outcomes following the new generation DES implantation. However, the use of ticagrelor for patients with ESRD should be carefully considered since it may increase the risk of all-cause or non-cardiac death with bleeding.

Patients with ACS and CKD tend to be excluded from randomized clinical trials on the effects of antiplatelet therapy, resulting in little evidence on outcomes associated with the type and duration of DAPT. Furthermore, although patients with advanced CKD, including those with ESRD, are at high risk of ischemia and bleeding (15, 16), there is little data on the suitable antiplatelet therapy for those undergoing DES implantation. The present study evaluated the ischemic and bleeding outcomes in this patient group.

The prevalence of ACS in patients with CKD is higher than that in patients with preserved renal function (17). When CKD is associated with co-morbidities, such as hypertension, diabetes, and dyslipidemia, calcium and phosphorus homeostasis is altered, and vascular calcification, including coronary artery atherosclerosis, is aggravated (18). Furthermore, changes in coagulation cascades, endothelial injury, and platelet reactivity worsen with higher platelet susceptibility to thrombin, increasing ischemic risks as CKD progresses (15). Meanwhile, patients with CKD are at a higher risk of bleeding owing to platelet dysfunction and abnormalities associated with increased systemic inflammation and oxidative stress triggered by endothelial dysfunction (19, 20). In particular, patients with advanced CKD present with higher rates of ischemic and bleeding events than do their counterparts with early disease (21). In the present study, the rates of MACCEs or bleeding events differed among patients with different disease stages; patients with ESRD had the highest rates of MACCEs and bleeding events, which translated into a high rate of net adverse clinical events relative to that observed in patients with CKD stage III/IV.

Ticagrelor is a potent P2Y_12_ inhibitor, developed to overcome the limitations of clopidogrel, such as low potency of platelet inhibition and a wide individual variability, leading to a high on-treatment platelet reactivity. However, studies involving ticagrelor are rare in ACS patients with CKD who have a higher rate of high on-treatment platelet reactivity associated with thrombotic ischemic events and cardiovascular death after PCI (22). In a subgroup analysis of the PLATO study, the benefit of ticagrelor was pronounced, including a larger absolute ischemic risk reduction in patients with CKD (eGFR of <60 mL/min) than in those with normal renal function; however, the corresponding risk of bleeding increased with CKD stage (23). In this trial, patients with ESRD requiring dialysis were excluded, and lesions were treated with the implantation of first-generation DESs, which are not commercially available. A recent study based on the SWEDEHEART database, compared the outcomes associated with clopidogrel and those associated with ticagrelor in patients having ACS and CKD, showing that patients with CKD stage III treated with ticagrelor had a lower mortality rate, MI, and stroke at 12 months than did their counterparts; however, no definite benefit was observed for patients with advanced CKD and ESRD. Meanwhile, among patients with CKD stage III, bleeding rates were similar in both treatment groups; however, a trend toward higher bleeding rates was observed in patients with advanced CKD, including those with ESRD. Findings from the interaction analysis were non-significant (24).

Analyses of our data from East Asian ACS patients with CKD of any stage have shown no significant differences in the 12-month rates of MACCEs or bleeding events between patients treated with ticagrelor- and those treated with clopidogrel-based DAPT. However, in patients with CKD stage III/IV without ESRD, ticagrelor-based DAPT was significantly associated with a reduced risk for MACCEs without any increase in the bleeding risks compared with clopidogrel-based DAPT. In the present analysis of patients with ESRD, the ticagrelor-based DAPT group did not present with any decrease in the risk of ischemic or bleeding events, but rather presented with numerically higher MACCEs or bleeding event rates. Despite bleeding risks, ticagrelor-based DAPT may be considered for ACS patients with CKD stage III/IV without ESRD. However, this approach requires verification in long-term randomized clinical trials that include mortality among their outcomes of interest.

This study has some limitations. First, it was not a randomized study; thus, selection bias may be present despite propensity score matching for as many variables as possible. Additionally, the unmeasured baseline and angiographic data may lead to residual confounding by an indication in the observational study. Second, the present registry-based study was not designed to compare the prognosis associated with the use of ticagrelor- vs. clopidogrel-based DAPT in patients with CKD. Therefore, the findings from this observational study cannot be applied to establish causal relationships, and persistent residual confounding factors should be considered in the interpretation of our results, although we tried to minimize the bias through propensity score matching. Third, this study did not assess the effect of pretreatment with an antiplatelet agent, which may affect the clinical outcomes. Fourth, as patients who experienced in-hospital events were excluded from this study, the early effects of antiplatelet therapy for CKD were not evaluated. Additionally, patients who underwent complex PCI or presented with ST-elevation MI could have been more frequently excluded from the study due to higher in-hospital event rate, providing a potential source of bias, especially in eGFR. Fifth, this study included East Asians who are more susceptible to bleeding events, and it could be difficult to apply the results of the study generally to the Western populations. Sixth, the follow-up period was relatively short to investigate the long-term outcomes in this patient group. Further, given the retrospective nature of the study, bleeding events not requiring hospitalization could have been missed. Seventh, this study did not include patients who used prasugrel for DAPT. Prasugrel vs. ticagrelor or clopidogrel for CKD patients should be investigated in future research. Finally, the side effects of P2Y12 inhibitors were not evaluated; similarly, the consequences of switching DAPT therapy types were not examined.

In conclusion, ticagrelor-based DAPT is associated with a lower rate of MACCEs than clopidogrel-based DAPT in safely discharged patients with ACS and CKD stage III/IV without ESRD, who underwent a new-generation DES implantation. However, the ticagrelor-based DAPT suggests increases in bleeding risk; therefore, a large size randomized study is required in the future to evaluate these risks and benefits.

## Data Availability Statement

The original contributions presented in the study are included in the article/Supplementary Material, further inquiries can be directed to the corresponding author/s.

## Ethics Statement

The studies involving human participants were reviewed and approved by Severance Hospital Human Research Protection Center IRB. The patients/participants provided their written informed consent to participate in this study.

## Author Contributions

JR and B-KK contributed to conception and design of the study and wrote the first draft of the manuscript. JR and S-JL organized the database and performed the statistical analysis. B-KK, S-JH, H-YK, C-MA, D-KC, J-SK, Y-GK, DC, M-KH, and YJ contributed to supervision, review, and editing. All authors contributed to manuscript revision, read, and approved the submitted version.

## Conflict of Interest

The authors declare that the research was conducted in the absence of any commercial or financial relationships that could be construed as a potential conflict of interest.

## Publisher's Note

All claims expressed in this article are solely those of the authors and do not necessarily represent those of their affiliated organizations, or those of the publisher, the editors and the reviewers. Any product that may be evaluated in this article, or claim that may be made by its manufacturer, is not guaranteed or endorsed by the publisher.
