# Supplementary material for: Ticagrelor vs. Clopidogrel in Acute Coronary Syndrome Patients With Chronic Kidney Disease After New-Generation Drug-Eluting Stent Implantation
Source: Front Cardiovasc Med. 2022 Jan 10;8:707722. doi: 10.3389/fcvm.2021.707722 (PMC8785207; doi:10.3389/fcvm.2021.707722)
Supplement: Supplementary file 1 [file Table_1.DOCX]

Supplementary Material

**Supplement 1. Clinical outcomes of the patients with CKD stage III/IV versus ESRD for twelve-month**

|  | **Total (N=1067)** | **CKD stage III/IV**  **(N=818)** | **ESRD**  **(N=249)** | **HR (95%CI)** | **P value** |
| --- | --- | --- | --- | --- | --- |
| MACCE | 87 (8.2%) | 46 (5.6%) | 41 (16.5%) | 3.11 (2.04-4.74) | <0.001 |
| Bleeding events (BARC type 3 or 5) | 52 (4.9%) | 33 (4.0%) | 19 (7.6%) | 1.99 (1.13-3.49) | 0.022 |
| Net adverse clinical events | 123 (11.5%) | 69 (8.4%) | 54 (21.7%) | 2.77 (1.94-3.96) | <0.001 |
| ***Individual event*** |  |  |  |  |  |
| All-cause death | 74 (6.9%) | 37 (4.5%) | 37 (14.9%) | 3.50 (2.22-5.53) | <0.001 |
| Cardiac death | 39 (3.7%) | 16 (2.0%) | 23 (9.2%) | 4.95 (2.61-9.36) | <0.001 |
| Non-cardiac death | 35 (3.2%) | 21 (2.5%) | 14 (5.7%) | 2.34 (1.19-4.60) | 0.014 |
| Myocardial infarction | 47 (4.4%) | 21 (2.6%) | 26 (10.4%) | 4.26 (2.39-7.58) | <0.001 |
| Stent thrombosis | 17 (1.6%) | 9 (1.1%) | 8 (3.2%) | 2.96 (1.14-7.67) | 0.026 |
| Cerebrovascular accident | 23 (2.2%) | 18 (2.2%) | 5 (2.0%) | 0.92 (0.34-2.48) | 0.870 |
| Ischemic | 13 (1.3%) | 11 (1.3%) | 2 (0.8%) | 0.60 (0.13-2.73) | 0.512 |
| Hemorrhagic | 10 (0.9%) | 7 (0.9%) | 3 (1.2%) | 1.42 (0.37-5.48) | 0.614 |
| Target-vessel revascularization | 39 (3.7%) | 22 (2.7%) | 17 (6.8%) | 2.61 (1.39-4.92) | 0.003 |
| BARC type 2 | 22 (2.1%) | 11 (1.3%) | 11 (4.4%) | 3.48 (1.51-8.04) | 0.003 |
| BARC type 2, 3 or 5 | 74 (6.9%) | 44 (5.4%) | 30 (12.0%) | 2.36 (1.48-3.76) | 0.001 |

Values are number of events (%) ESRD = end staged renal disease; MACCE = major adverse cardiac and cerebrovascular events; BARC = Bleeding Academic Research Consortium

**Supplement 2. Clinical Outcomes of the patients with ESRD for twelve-month between the ticagrelor- and the clopidogrel-based DAPT group**

|  | **Total (N=249)** | **Ticagrelor**  **-based DAPT**  **(N=77)** | **Clopidogrel**  **-based DAPT (N=172)** | **HR (95%CI)** | **P value** |
| --- | --- | --- | --- | --- | --- |
| MACCE | 41 (16.5%) | 16 (20.8%) | 25 (14.5%) | 1.49 (0.80-2.80) | 0.211 |
| Bleeding events (BARC type 3 or 5) | 19 (7.6%) | 8 (10.4%) | 11 (6.4%) | 1.75 (0.48-6.20) | 0.401 |
| Net adverse clinical events | 54 (21.7%) | 22 (28.6%) | 32 (18.6%) | 1.64 (0.96-2.83) | 0.073 |
| ***Individual event*** |  |  |  |  |  |
| All-cause death | 37 (14.9%) | 18 (23.4%) | 19 (11.0%) | 2.25 (1.18-4.29) | 0.013 |
| Cardiac death | 23 (9.2%) | 10 (13.0%) | 13 (7.6%) | 1.78 (0.78-4.05) | 0.172 |
| Non-cardiac death | 14 (5.7%) | 8 (10.4%) | 6 (3.4%) | 3.17 (1.10-9.14) | 0.033 |
| Myocardial infarction | 26 (10.4%) | 13 (16.9%) | 13 (7.6%) | 2.39 (1.11-5.16) | 0.026 |
| Stent thrombosis | 8 (3.2%) | 3 (3.9%) | 5 (2.9%) | 1.37 (0.33-5.72) | 0.668 |
| Cerebrovascular accident | 5 (2.0%) | 1 (1.3%) | 4 (2.3%) | 0.56 (0.06-5.04) | 0.608 |
| Ischemic | 2 (0.8%) | 0 (0%) | 2 (1.1%) | 0.03 (0.001-56.7) | 0.579 |
| Hemorrhagic | 3 (1.2%) | 1 (1.3%) | 2 (1.2%) | 0.52 (0.06-4.67) | 0.561 |
| Target-vessel revascularization | 17 (6.8%) | 7 (9.1%) | 10 (5.8%) | 1.61 (0.61-4.24) | 0.332 |
| BARC type 2 | 11 (4.4%) | 6 (7.8%) | 5 (2.9%) | 2.83 (0.86-9.27) | 0.086 |
| BARC type 5 | 9 (3.6%) | 4 (5.2%) | 5 (2.9%) | 1.93 (0.52-7.17) | 0.329 |
| BARC type 2, 3 or 5 | 30 (12.0%) | 14 (18.2%) | 16 (9.3%) | 2.10 (1.03-4.31) | 0.042 |

Values are number of events (%) ESRD = end staged renal disease; MACCE = major adverse cardiac and cerebrovascular events; BARC = Bleeding Academic Research Consortium

**Supplementary Figure Legends**

| **Supplementary Figure 1.** | **Subgroup analyses of major bleeding events between the Ticagrelor- and the clopidogrel-based DAPT groups**  DAPT = Dual Anti Platelet Therapy |
| --- | --- |
| **Supplementary Figure 2.** | **Twelve-month cumulative incidence of major adverse cardiac and cerebrovascular (A), bleeding (B), or net adverse clinical (C) events on ESRD**  ESRD = End Stage Renal Disease |
