# Supplementary figures and images for: Ticagrelor vs. Clopidogrel in Acute Coronary Syndrome Patients With Chronic Kidney Disease After New-Generation Drug-Eluting Stent Implantation
Source: Front Cardiovasc Med. 2022 Jan 10;8:707722. doi: 10.3389/fcvm.2021.707722 (PMC8785207; doi:10.3389/fcvm.2021.707722)

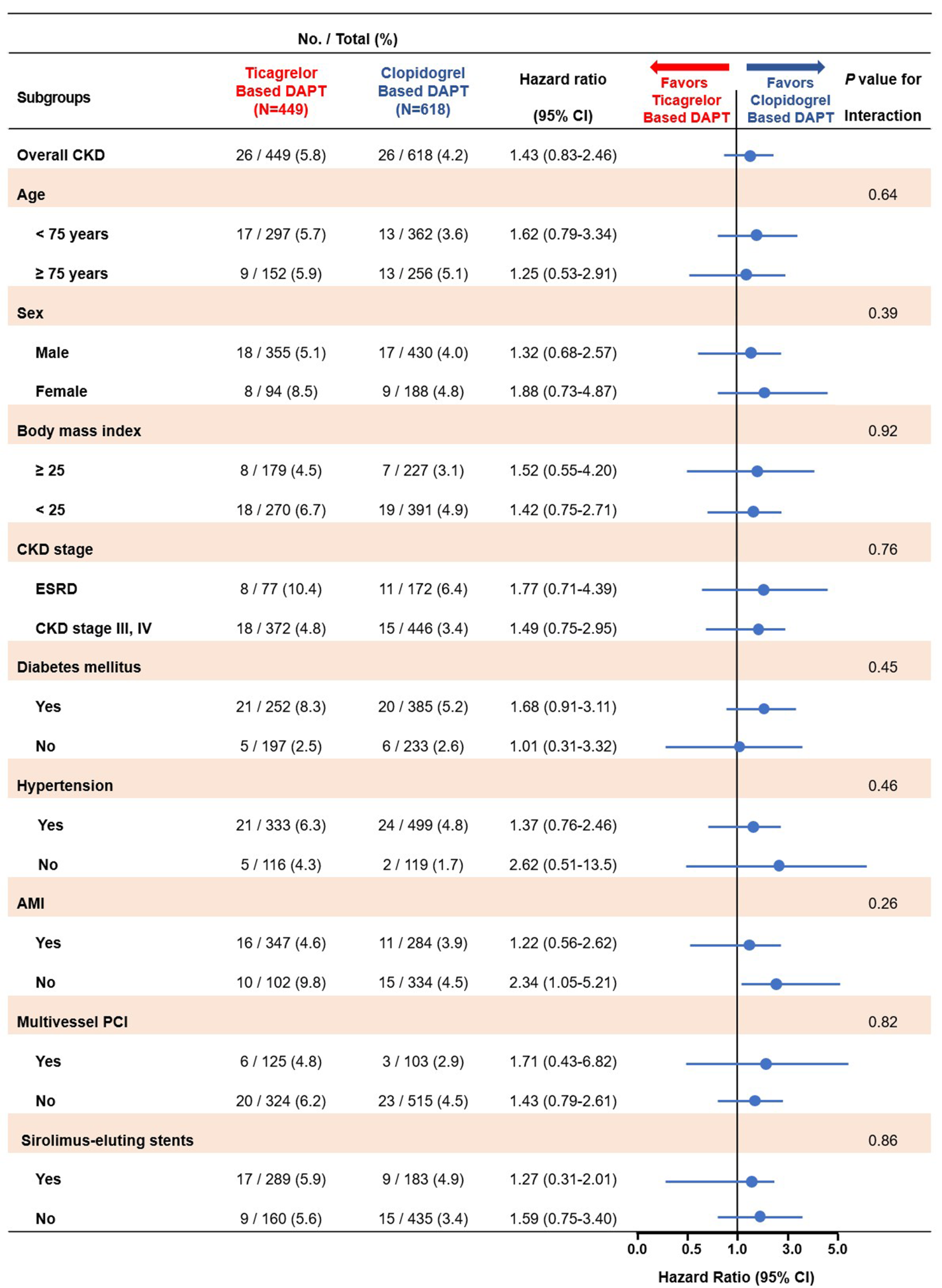

Supplement: Supplementary file 2 [file Image_1.TIF]

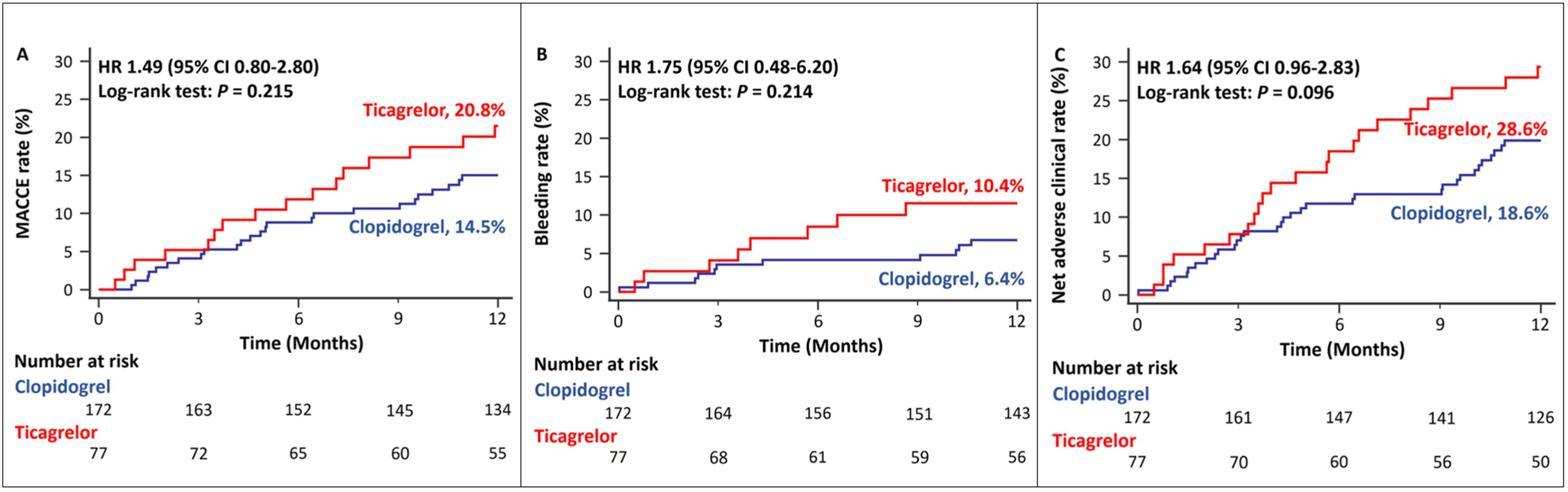

Supplement: Supplementary file 3 [file Image_2.TIF]
